# A Low-Cost, Self-Made Simulation Model for Shoulder Arthroscopy Surgical Skills Acquisition

**DOI:** 10.7759/cureus.107049

**Published:** 2026-04-14

**Authors:** Thomas A Naylor

**Affiliations:** 1 Trauma and Orthopaedics, Manchester University NHS Foundation Trust, Manchester, GBR

**Keywords:** orthopaedic arthroscopy, orthopaedic registrar training, orthopedic residency, shoulder and knee arthroscopy, shoulder sport, simulation in medical education, surgery simulation

## Abstract

The benefits of simulation training for technical skills development in orthopaedic arthroscopic surgery have been well documented. Access and high costs are barriers to regular use, which may be overcome with a low-cost, self-made model. Examples from established knee arthroscopy models can be adapted to a shoulder arthroscopy model for training specific skills.

Utilising an inspection endoscope camera, smartphone, shoebox, and easily accessible materials, a self-made shoulder arthroscopy simulator can be constructed for £30. Instructions for design and construction are included, with interchangeable viewing and instrumentation portals.

A simulation working environment is created with exercises that can be used to develop specific skills required for arthroscopy. The use of a simulated humeral head and checkpoints within the working space develops efficiency in navigation through the shoulder joint. Exercises in manoeuvring elastic bands around screw hooks challenge bimanual dexterity and triangulation from different angles. Fine control and visuospatial processing are further developed with object tracing exercises.

Residents can create their own low-cost simulation boxes at home, and the exercises described aim to improve the acquisition of specific surgical skills in shoulder arthroscopy. The practical applications and value of the shoulder shoebox endoscope simulator for orthopaedic residents in training would benefit from validation.

## Introduction

Shoulder arthroscopy is a widely used technique in upper limb orthopaedics for minimally invasive operations, including rotator cuff repair, shoulder stabilisation, arthrolysis, and decompression. Arthroscopy, much like laparoscopy, has a significant learning curve that requires multiple hours of practice and exposure to build and maintain skills [1].

Efficient and safe arthroscopic operating relies on hand-eye coordination, triangulation of instruments, and visuospatial processing of 3D spaces from a 2D image [2]. Compared to arthroscopy of the knee, the more frequent use of multiple different viewing portals at oblique angles with pronounced fulcrum effects and challenging bimanual triangulation at angles convergent with or directly opposing the viewpoint of the operator adds to technical difficulties [3].

Simulator models and virtual reality trainers have a clear role in counteracting limited theatre time and shoulder subspecialty operating exposure. High-fidelity models have been shown to be effective in improving skills, but these can be lost within 6 months of not operating [4]. Maintaining skills with the use of these simulators is made difficult due to the prohibitive costs of £250 to £140,000 [5] and the lack of continuous access.

The objective of this project was to construct a simulation model for the lowest cost possible and to explore and design exercises for the development of arthroscopic technical skills. The aim of this project was to establish proof of concept of a low-cost, easily constructable, and self-made shoulder arthroscopy simulation model to allow surgical residents to acquire and maintain the technical skills required for these challenging procedures at home.

## Technical report

Portable arthroscopic boxes have been described previously and demonstrated as improving skills in knee and shoulder arthroscopy [5,6]. The Cigar box arthroscopy model is well recognised by orthopaedic residents and validated [7]. There are other examples described for shoulder arthroscopy using polypropylene boxes, 3D-printed materials, and rubber casting, and polyethylene tubing with webcams [5,8,9]. The use of self-made training cameras has been shown to be as beneficial as commercially available models [10].

**Figure 1:**
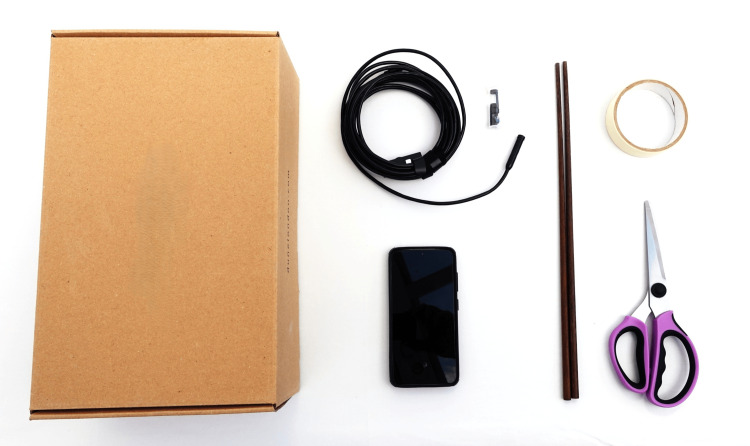
Basic components for construction of the shoulder shoebox endoscope simulator The simplest version of the simulation model requires a shoebox (or equivalent), commercially available USB endoscope inspection camera, cooking chopsticks, tape, scissors.

This model takes advantage of the availability of cheap inspection endoscope cameras. The shoulder joint itself is simulated by a segmented shoebox, with instruments in the form of long cooking chopsticks or wooden dowels of 4 mm diameter. Required equipment for the basic model setup is shown in Figure 1. 

Various inspection endoscope cameras are available from online and in-person retailers, with the key features required being LED lighting circumscribing a wide-angle lens on a semi-rigid rod. The camera is connected to the user’s smartphone, which can be placed at a convenient place above or behind the simulation box as the viewing screen, and also allows recording and review of practice via screen capture features.

**Figure 2:**
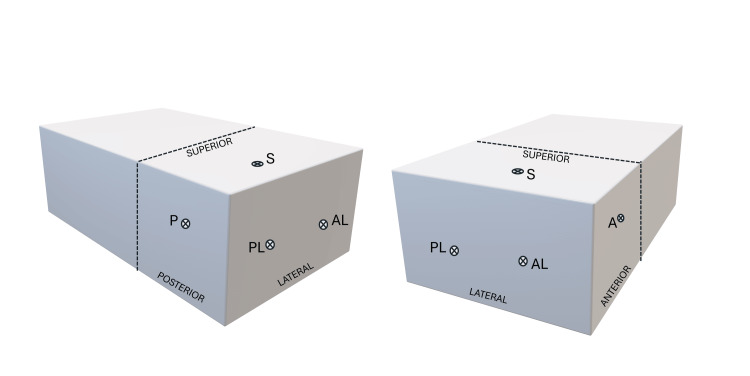
Example shoulder portals for simulation training Possible portal placement, for an example right shoulder. Complete flexibility possible in portal placement with design and choice dependent on the user.

A 90-degree bend is applied to the proximal end of the rod, allowing the user to orient what is “north” and “south” on the camera screen. Additionally, a bend can be applied to the distal end of the camera rod to recreate a 30- or 70-degree viewing angle, as is standard for shoulder arthroscopes. Portals can be placed in various locations of the model box, similar to port-on-demand techniques used in real arthroscopy, with suggestions provided in Figure 2.

The box can be opened to set up various exercises and supplementary equipment, and flipped between being a ‘left’ and ‘right’ shoulder to improve adaptability and focus on weaknesses in a particularly unidextrous resident.

## Discussion

**Figure 3:**
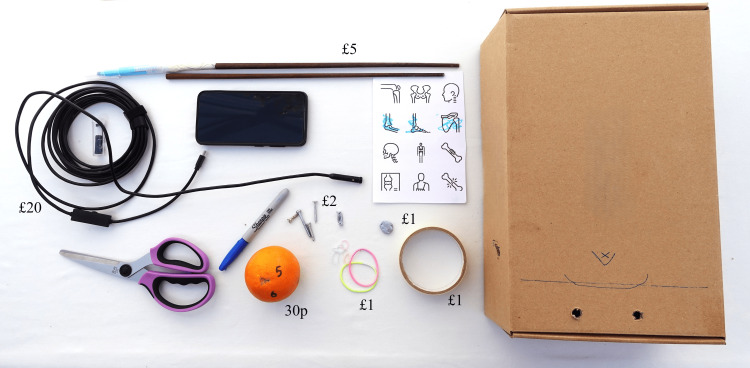
Total costs for all components of the shoulder shoebox arthroscopy simulator Additional components required for exercises as described, including screws, rubber bands (hairbands), an orange (or other spherical object), and a marker pen.

The total cost for all parts of the shoulder shoebox endoscope simulator box, with the exclusion of the user’s smartphone, would have been £30.30, although most materials were available already from around the author’s home environment without additional purchasing required (Figure 3). 

The model recreates the viewing points and movement patterns of an in vivo shoulder arthroscopy, albeit with low fidelity. However, the real benefits of the simulator model come from exercises that can be used for specific skill acquisition.

**Figure 4:**
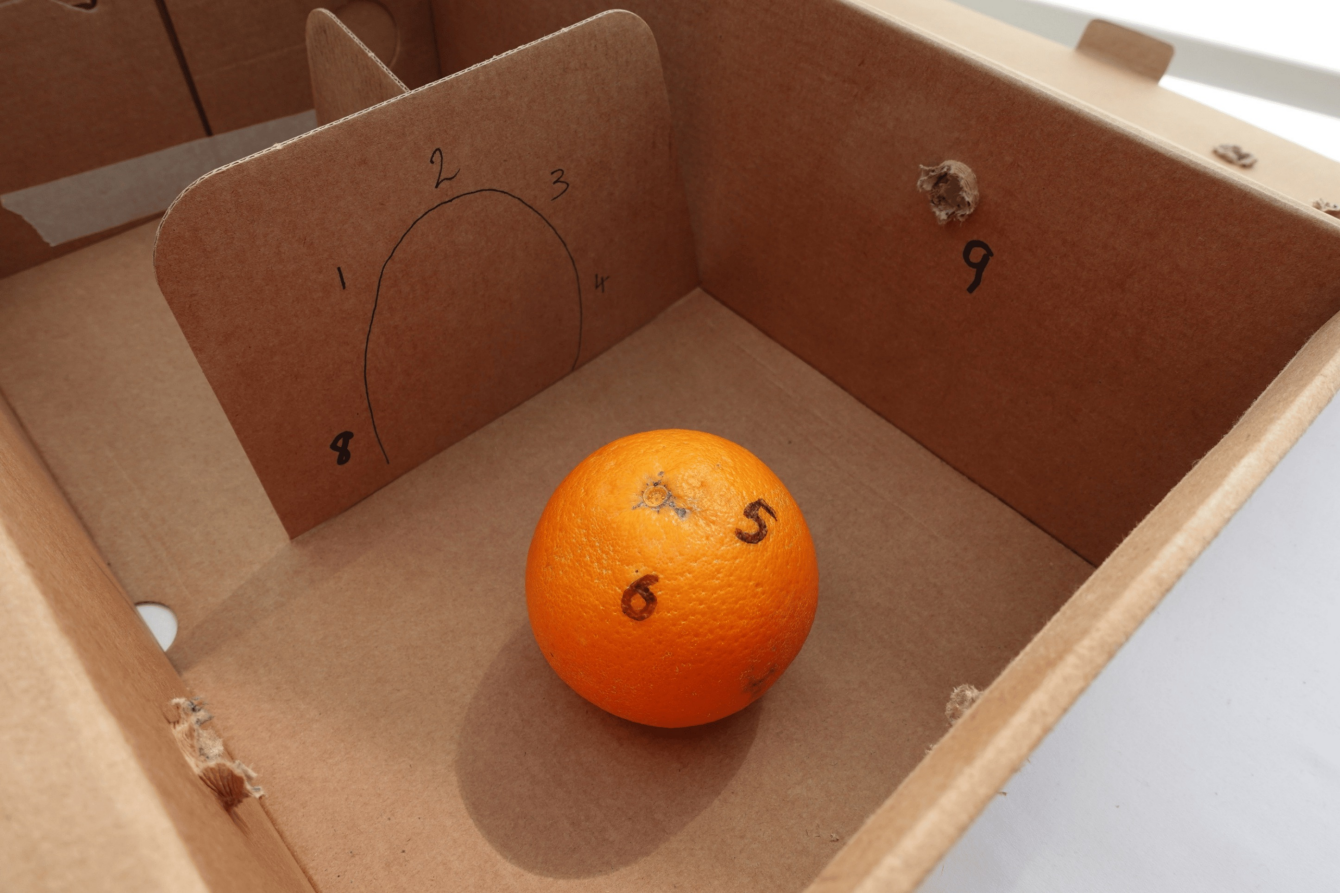
Simulation exercise 1 An orange or other spherical object representative of the humeral head with numbered check-points labelled throughout should be navigated in order, recreating stepwise progression through a diagnostic shoulder arthroscopy.

The first exercise aims to establish a set routine for the user to follow for diagnostic arthroscopy. An orange is used to replicate the humeral head, with points marked and numbered within the box (Figure 4).

The user must check off each number, which correlates with anatomical points visualised in every diagnostic arthroscopy, e.g. subscapularis tendon, superior labrum and long head of biceps insertion, rotator cuff insertion, posterior and anterior labrum. With repetition, the user develops movement patterns and efficiency in navigating through the shoulder. Demonstration of this exercise can be found in Supplementary Material Video 1. 

**Video 1:** Demonstration of simulation exercise 1

With industry partnerships, this model would also allow practice of suture anchor placements within the orange ‘humeral head’.

**Figure 5:**
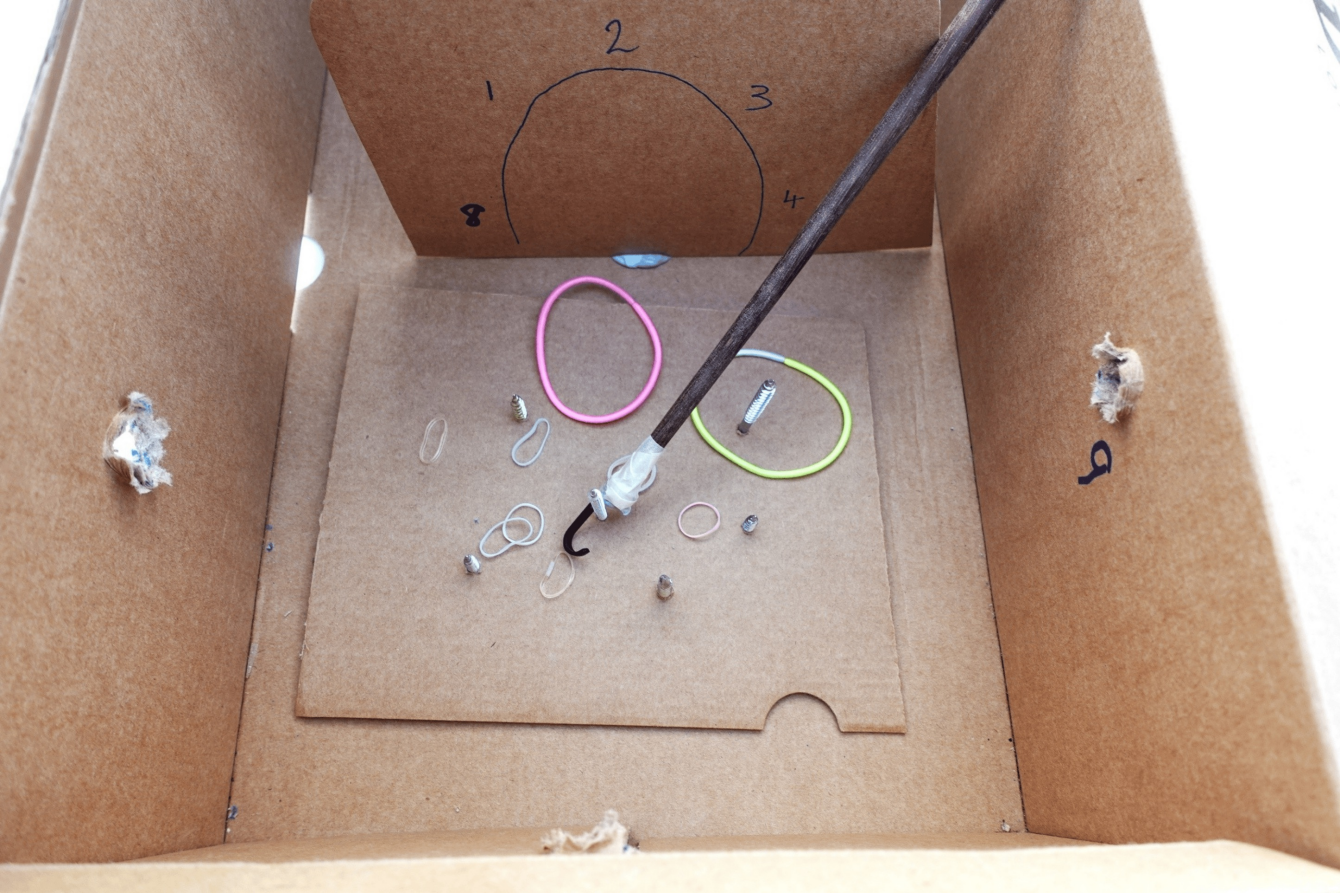
Components of simulation exercise 2 Use of screws of various diameters and lengths, along with a small instrumentation hook and elastic bands for practice of instrument handling, dexterity, and visuospatial processing

The second exercise focuses on developing the skills required to transition and adapt to different angles of triangulation, dependent on the direction in which the viewing portal and instrumentation meet, as well as bimanual coordination, e.g., anterior-posterior, posterior-lateral, and lateral-anterior. It also fine-tunes rotational movements of instrumentation required in the passage of sutures in stabilisation and cuff repair procedures. An additional piece of card penetrated with screws of varying lengths and diameters is inserted in the box working area, accompanied by elastic bands of different sizes (in the author’s example, various children’s hair ties are used; seen in Figure 5).

Many endoscope cameras come with a hook attachment that is applied to the instrument rod and is used to pick up and loop the bands over the screws. An example of this exercise is available as Supplementary Material Video 2.

**Video 2:** Demonstration of simulation exercise 2

A timer can be set and progress tracked as the user becomes more proficient in the exercise. Additional restrictions can be applied to challenge the user by allowing only specific portals to be used.

The final exercise challenges fine control of the instrumentation with simple graphics placed in the box working area. A pen is attached to the end of the instrumentation rod, and the user must trace and colour in the shapes neatly, replicating the controlled and accurate movement required when using arthroscopic ablation wands and shavers in many procedures. Demonstration of this exercise can be found in Supplementary Material Video 3. 

**Video 3:** Demonstration of simulation exercise 3

This simulation model was demonstrated at a regional orthopaedics resident simulation competition day and received excellent feedback from the faculty and residents in attendance. The short- and long-term benefits of the model require validation, and efforts are in progress to complete this. There is potential for further development and adaptations to more closely simulate arthroscopy of other joints, e.g., the elbow, wrist, and hip.

## Conclusions

This project has demonstrated the ease with which a low-cost shoulder arthroscopy simulator model can be self-made with readily available materials. The instructions included will allow other residents and training programmes to create their own simulation boxes at home. Simulation exercises described aim to improve the acquisition of specific surgical skills in shoulder arthroscopy. The practical applications and value of the shoulder shoebox endoscope simulator for orthopaedic residents in training would benefit from validation.
